# A twin pregnancy with partial hydatidiform mole and a coexisting normal fetus delivered at term: A case report and literature review

**DOI:** 10.1016/j.crwh.2023.e00544

**Published:** 2023-09-16

**Authors:** Alessandro Libretti, Daniela Longo, Stefano Faiola, Alberto De Pedrini, Libera Troìa, Valentino Remorgida

**Affiliations:** a Unit of High-Risk Pregnancy, department of Gynaecology and Obstetrics, University Hospital Maggiore della Carità, 20090 Novara, Italy; b School of Gynaecology and Obstetrics, University of Eastern Piedmont, 20090 Novara, Italy; c Fetal Therapy Unit 'Umberto Nicolini', Buzzi Children's Hospital, 20134 Milan, Italy; d Department of Woman, Mother and Neonate, Buzzi Children's Hospital, 20134 Milan, Italy

**Keywords:** Hydatiform mole, Partial mole, Living fetus, Case report

## Abstract

Hydatiform mole occurs in 1/1000 singleton and 1/20000–100,000 twin pregnancies. Although the pregnancy often ends in a miscarriage or presents with many obstetric complications such as preeclampsia, vaginal bleeding, hyperthyroidism, prematurity, or fetal malformations, in some cases of twin pregnancy, one of the fetuses can develop normally. Coexistence of a viable fetus in a twin molar pregnancy is more commonly described for cases of complete hydatiform moles than partial hydatiform moles. A partial hydatiform mole coexisting with a normal fetus was suspected in a 40-year-old woman, G2P1, at twelve weeks of gestation of a twin dichorionic diamniotic pregnancy. Serial antenatal ultrasound scans and serial evaluations of human chorionic gonadotropin were performed, and a healthy baby was delivered at term without any obstetric or neonatal complications.

A twin pregnancy with partial hydatidiform mole and a coexisting normal fetus is a rare obstetric condition that can result, under proper management, in the delivery of a healthy baby without any sequelae for the mother or child.

## Highlights

• Hydatiform mole is a very rare condition, especially for twin pregnancies.
• It often ends in a miscarriage or presents many obstetrics complications.
• Hydatiform mole can progress to gestational trophoblastic neoplasia.
• The evaluation of beta human chorionic gonadotropin (BhCG) is crucial in the diagnosis and the management of hydatiform mole.
• A twin pregnancy with coexistence of mole and fetus can result in a healthy baby.

## Graphical abstract

A 40-years-old, Caucasian, G2P1 woman which pregnancy was complicated by a partial hydatiform mole and a coexisting normal fetus, succeded, under proper management, to have a vaginal delivery at term and a healthy baby, without any obstetrical nor neonatal complications.

Moreover, the pregnancy was completely uneventful and no evidence of persistent trophoblastic disease was noticed at follow-up.

**Figure:**
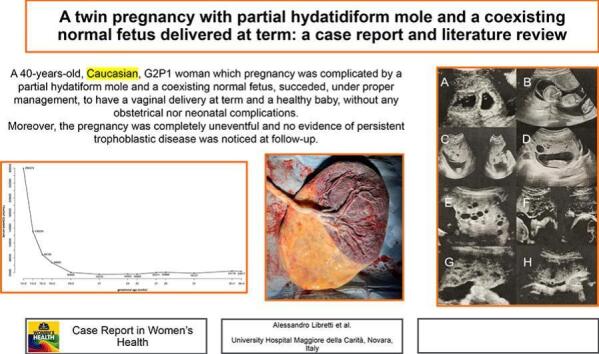

## 1 Introduction

Hydatiform mole (HM), also known as molar pregnancy, is the most common form of gestational trophoblastic disease [1]. It represents a premalignant form of gestational trophoblastic neoplasia (GTN) [1,2]. The incidence of HM varies around the world from 1 to 2 per 1000 pregnancies in North America and Europe to 10 per 1000 in India and Indonesia [1,2]. The molar pregnancy may be classified as complete or partial, which differ by gross morphology, histopathology, karyotype, and risk of malignancy [3]. A twin pregnancy with HM and a living fetus is an even rarer entity, with an incidence of 1 in 20,000 to 100,000 pregnancies [1,2]. There are two types of these twin pregnancies: those with a complete hydatidiform mole and a coexistent fetus (CHMCF) and those with a partial hydatidiform mole and a coexistent fetus (PHMCF) [4,5] In the case of twins with the hydatiform mole coexisting with a living fetus, the placental mass is differentiated into a normal placenta connected to a normal fetus and a molar placenta [4].

Considering the possible complications, such as preeclampsia, hemorrhage and hyperthyroidism, and the risk of developing GTN, in the cases of twin molar pregnancy, women should be appropriately counselled about the risks of continuing with the pregnancy and offered the option of termination [6].

## 2 Case Presentation

A 40-year-old woman, G2P1, had her first obstetric check at 5 weeks and 5 days of pregnancy as per her desire. The pregnancy was conceived spontaneously. She had had an uneventful pregnancy and a vaginal delivery three years earlier. Her past medical history included Hashimoto's thyroiditis and favism. She had never had surgery and was a non-smoker.

**Fig. 1:**
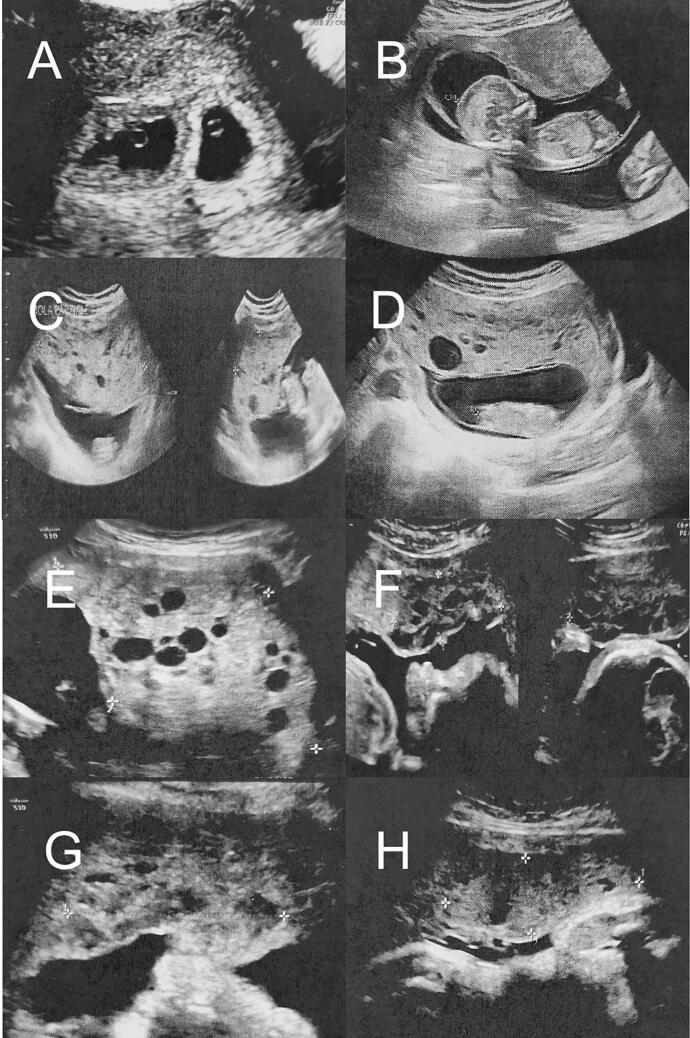
Ultrasound findings per gestational age. A Dichorial-diamniotic pregnancy (5 + 5 weeks of pregnancy). B Living fetus (13 + 1 weeks of pregnancy) with normal placenta. C Honeycomb like placental mass 10.4 × 4.5 × 4.7 cm (13 + 1 weeks of pregnancy). D Honeycomb like placental mass 10 × 10 × 3.5 cm (15 + 4 weeks of pregnancy). E Honeycomb like placental mass 11.2 × 6.6 cm (17 + 2 weeks of pregnancy). F Honeycomb like placental mass 6.8 × 5.9 × 2.5 cm (25 + 1 weeks of pregnancy). G Honeycomb like placental mass 8.2 × 5.7 × 2.2 cm (31 weeks of pregnancy). H Honeycomb like placental mass 7.8 × 7.9 × 2.6 cm (37 weeks of pregnancy).

Neither mother nor father reported any significant family history, in particular of hydatiform mole or twin pregnancy. The first ultrasound scan (US) (5 + 5 weeks of pregnancy) revealed a dichorionic diamniotic pregnancy (Fig. 1, A). Blood tests (hemoglobin, hematocrit, white blood cells, platelets, liver, and kidney's function markers) were normal. A second US performed at 7 + 2 weeks of pregnancy showed two embryos (the first measuring 12.9 mm, and the second measuring 14.4 mm), both with cardiac motion.

During the first-trimester screening, performed at 12 weeks of pregnancy, one fetus had a crown–rump length (CRL) of 65 mm and a normal nuchal translucence (1.7 mm). Its placenta had a normal appearance. The other fetus no longer had cardiac motion, and its placenta appeared vacuolated (like Swiss cheese or snowstorm in appearance) hinting at a hydatiform mole. No other abnormalities were found during this exam.

At this point the parents were counselled about the risk of maternal complications and progression in GTN, but they decided to continue with the pregnancy and refused any further tests, including karyotype study.

**Fig. 2:**
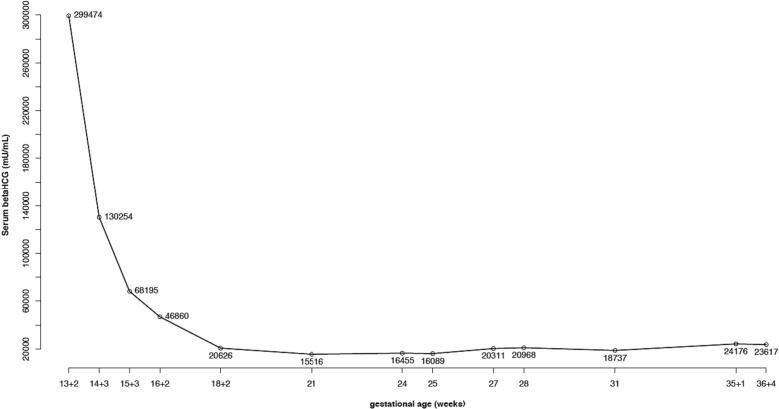
Beta human Chorionic Gonadotropin (BhCG) trend for gestational age.

US follow-up for the assessment of both the growth of the living fetus and the size of the molar mass, together with serial beta human chorionic gonadotropin (BhCG) samples were then scheduled*.* The BhCG trend for gestational age is shown in Fig. 2*.* The rest of the pregnancy was completely uneventful, with a regression of the molar mass and a decrease in the BhCG level over the weeks, together with regular growth of the living twin (Fig. 1).

**Fig. 3:**
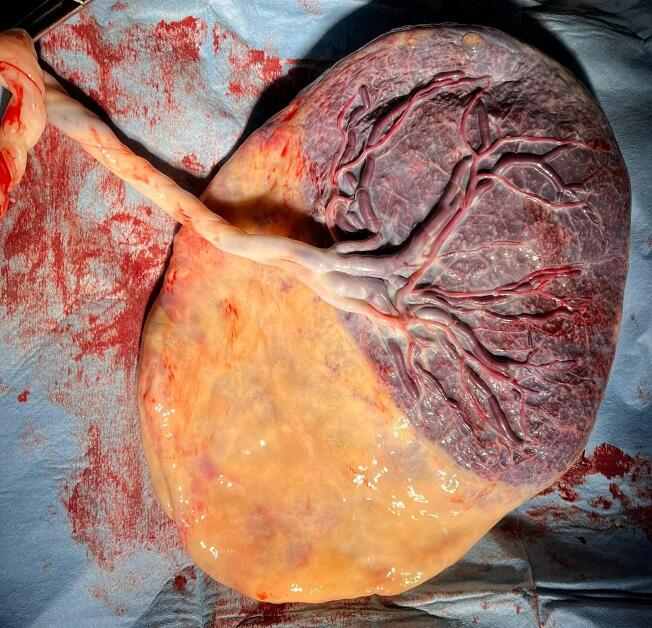
Macroscopic evaluation of the placenta: chorionic (fetal) plate.

**Fig. 4:**
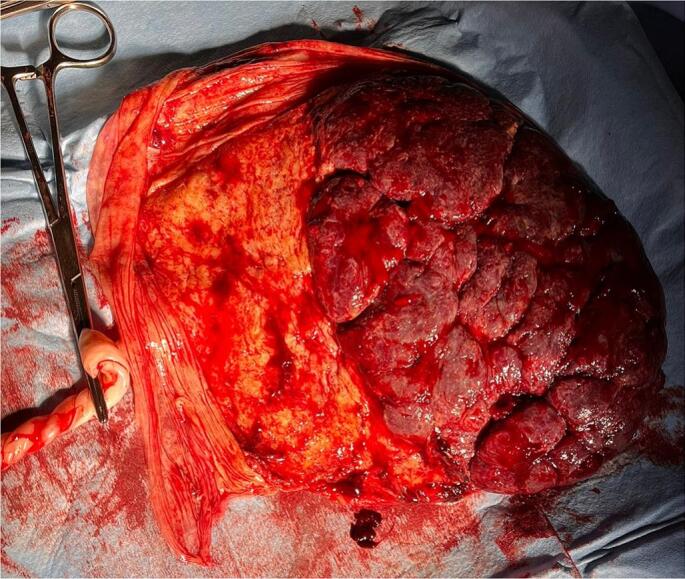
Macroscopic evaluation of the placenta: basal (maternal) plate.

The woman delivered vaginally at 38 + 1 weeks of gestation, after 8 h of active, spontaneous, labor. The baby (2710 g) was in good health (APGAR score 9–9) and his first neonatological evaluation revealed no abnormalities. The placenta, together with the molar mass, were spontaneously evacuated 4 min after birth (Fig. 3, Fig. 4). The blood loss at the delivery was 200 cc. The histopathological examination confirmed the partial hydatiform mole and normal placenta. Maternal BhCG samples were collected every 4 weeks during the post-partum period until 2 normal values were obtained. The first sample collected was already negative. No evidence of persistent trophoblastic disease, of evolution to neoplasia or of lung metastases was noted at the 6-month follow-up.

## 3 Discussion

HM is classically associated with several pregnancy complications, such as spontaneous abortions, intrauterine deaths, hyperthyroidism and preeclampsia, and patients are usually advised to terminate the pregnancy, in order to limit the risk of progression to GTN. [7]. The risk of progression to post-molar neoplasia is about 15–20% of cases in CHM and 1.5% in PHM [2]. In CHM, ultrasonography shows diffuse cystic spaces within the placenta, an increase in the diameter of the gestational sac and no embryo or fetus [3]. In the case of PHM, fetal elements can be present although rarely, with or without fetal cardiac motion, because of early demise after implantation [3]. Focal hydatidiform swelling of chorionic villi, irregularity and increased echogenicity of the decidua or placenta are US characteristics of PHM [3]. The evaluation of BhCG is crucial in the diagnosis of HM, but it does not help in differentiating between CHM and PHM [8]. Furthermore, its application is very limited in the case of twin pregnancies [8]. That said, antenatal US together with postnatal histopathological exams play a key role in the diagnosis [8]. The differential diagnosis of PHM includes CHM and hydropic spontaneous abortion [8]. Histologic examinations in all miscarriages/anembryonic sacs are needed to differentiate CHM or indeed PHM when a first-trimester US did not show any embryo or fetal structures [8].

A review of the literature on twin pregnancies with a PHM and a coexistent normal fetus, delivered live, was conducted. Entering the terms “twin pregnancy” and “mole” on PubMed, Medline, and Google Scholar (some of the main online search sources) generate 315 results. After the analysis of all titles and abstract, only studies in English, involving a twin pregnancy with the coexistence of PHM and a living fetus delivered live, were included. Eight papers on 9 cases [3,4,[9], [10], [11], [12], [13], [14]] were identified. All the information about the patient, the pregnancy, and the outcomes is presented in Table 1.

**Table 1:** Included articles in the literature review. Nine cases on twin pregnancies with partial hydatidiform mole and a co-existing normal fetus that ended with the delivery of a living newborn.

Reference	Age (years)	Parity	Type of conception	GA at diagnosis (weeks)	Pregnancy complications	Maximum peak of beta HCG levels mU/mL (GA)	Mode of delivery	GA at delivery (weeks)	Neonatal outcomes	BhCG negative (weeks)	Persistent GTD
Chu et al., 2004	29	G5P3	–	16	Vaginal bleeding, Preterm labor, fever	–	C/S	24 + 2	Living	–	No
Copeland et al., 2010	29	G4P3	–	8	Reflux nepropathy	–	C/S	28	Living	52	No
Rai et al., 2014	25	–	Ovulation induction	13	Vaginal bleeding, Pre-term labor	374,747 (13)	C/S	36	Living	2	No
Rathod et al., 2015	24	G2P1	–	13	Abdominal pain, Pre-term labor	121,993 (after delivery)	Vaginal	28	Living	4	No
Lin et al., 2021	33	G2P0	IVF-ET	24	Oligohydramnios	105,851 (24)	C/S	40	Living	109	Yes (GTN)
Liang et al., 2022	24	–	–	32	Pre-eclampsia, IUGR	126,203 (−)	Vaginal	32	Death (day 5)	–	No
Liang et al., 2022	30	–	–	26	Vaginal bleeding	61,110 (−)	C/S	34	–	–	Yes (GTN)
Tolcha et al., 2022	40	G13P10	S	28 + 6	Pre-eclampsia	215,400 (28 + 6)	C/S	29 + 6	Living	7	No
Xing et al., 2022	24	G4P3	S	9	None	–	C/S	36 + 6	–	–	No
Current case, 2023	40	G2P1	S	12	None	299,474 (13 + 2)	Vaginal	38 + 1	Living	8	No

(−): Missing.

S: Spontaneous.

GA: Gestational Age.

IUGR: intrauterine growth restriction.

BhCG: beta human chorionic gonadotropin.

C/S: cesarean section.

FU: follow-up.

GTN: gestational trophoblastic neoplasia.

IVF-ET: In vitro fertilization and embryo transfer.

The presenting symptoms were vaginal bleeding in three cases, preeclampsia in two cases; two reports did not state the presenting symptoms, and one case no symptoms at all. In one case the presenting symptom was abdominal pain. All cases but one had complications: preeclampsia (2 cases), intrauterine growth restriction (1 case), oligohydramnios (1 case), preterm labor (2 cases), fever (1 case), reflux nephropathy (1 case). Two cases progressed to gestational trophoblastic neoplasia. Two out of 9 pregnancies ended with a vaginal delivery. One of the newborns died, 5 days after the cesarean section, from respiratory distress syndrome.

As recommended by the Royal College of Obstetricians and Gynaecology in its guidelines for the management of gestational trophoblastic disease [15], all these women were counselled about the potential increased risk of perinatal morbidity and the risk of GTN, but they decided to continue their pregnancies.

The management of pregnancy in the case of PHM and the coexistence of a normal fetus can be very challenging, especially in the case of twins, partly because of the very limited number of cases. As advised in a previous review on PHM coexisting with a living fetus, when a woman decides to continue with the pregnancy, a multidisciplinary team of obstetrician, maternal fetal medicine specialist, gynecologic oncologist and neonatologist should be involved in the patient's care [16].

Moreover, an amniocentesis should be offered at 16 weeks to determine fetal karyotype [16]. US to rule out congenital abnormalities in the living fetus must be organized together with serial scans for the high risk of fetal growth restriction and oligohydramnios, at least every 2–3 weeks [16]. Patients are at risk of life-threatening hemorrhage at the time of delivery and obstetricians must be aware of this too [16]. As said, the patient needs to be monitored after delivery through BhCG samples, for early diagnosis and management of post-molar GTN [15].

## 4 Conclusion

Although very rare, the possibility of a twin pregnancy with partial hydatidiform mole and a coexisting normal fetus must be considered. Due to the potential challenges associated with such pregnancies, referral to a tertiary center is recommended. Patients who decide to continue with the pregnancy following counselling should be supported and managed by a multidisciplinary team.

Serial US, serial BhCG samples (including during the post-partum period), and strict surveillance during the post-partum period are essential, considering the risk of hemorrhage and persistent trophoblastic disease. The present case confirms the possibility of a good outcome and can help support the counselling and management of twin pregnancies with a PHM and coexistent normal fetus.
